# Utilizing a Mobile Clinical Trials Unit: The Good, The Bad, and The Ugly from Eight Years of Experience

**DOI:** 10.1002/alz70859_098225

**Published:** 2025-12-25

**Authors:** Anna Sladky, Jenny Echevarria Medina, Patricia Lowe, Rhiannon Capuano, Ijeoma Mba, Juris Janavs, Karen Colombo, Terrence LaFrance, Elisabeth Burnett, Heather Wynne‐Phillips, Ram J Bishnoi, Amanda G Smith

**Affiliations:** ^1^ USF Health Byrd Alzheimer's Institute, Tampa, FL USA

## Abstract

**Background:**

In 2016, we created a mobile clinical trials unit to increase enrollment in Alzheimer’s disease clinical trials across the Tampa Bay and surrounding areas in the state of Florida.

**Method:**

We reflected upon the successes and failures of the unit and reviewed revenue versus expenditures.

**Result:**

Positive findings include increased visibility in the community, positive feedback from participants, increased requests for clinic appointments and memory screenings, increased enrollment in certain trials, and national and international interest in expanding the concept.

Challenges over the lifetime of the mobile clinical trials unit include cost of moving the unit and need to use a trucking company to do so, space requirements for parking, unexpected maintenance issues and associated costs due to the age/wear‐and‐tear, hesitancy of sponsors to adopt the concept, FDA requirements for 1572s requiring a physical location, need for development of unit‐specific SOPs, staffing issues, impact of COVID‐19, and additional work and accountability transporting study materials such as drug, source documents, and labs. Between 2020 and 2024, our expenditures exceeded $100,000 while our revenue totaled $27,441.00. When the concept of the mobile clinical trials unit was formed, many of our clinical trials were using oral medications. As the research landscape changed, and we saw a surge of new trials testing monoclonal antibodies, we hit several barriers. Namely, the sponsors of these types of trials were unwilling to support use of the mobile clinical trials unit due to concerns with transporting the study drug, despite our efforts to alleviate these concerns through use of SOPs.

**Conclusion:**

There are both pros and cons associated with the operation of a mobile clinical trials unit, especially when the unit itself is only semi‐mobile. While the unit is ideal for furthering community outreach and for observational studies, the costs of moving and maintaining the unit without a consistent funding source and without consistent study visits to bring in revenue has resulted in the retirement of the mobile clinical trials unit at our site. Transferring the unit to another community engagement team within our institution was determined to be the best option for the future use of the unit.

